# Polymer-Salt Aqueous Two-Phase System (ATPS) Micro-Droplets for Cell Encapsulation

**DOI:** 10.1038/s41598-019-51958-4

**Published:** 2019-10-29

**Authors:** Mohammad Mastiani, Negar Firoozi, Nicholas Petrozzi, Seokju Seo, Myeongsub Kim

**Affiliations:** 1 Center for Biosignatures Discovery Automation, School of Electrical, Computer and Energy Engineering, Arizona State University, Tempe, AZ 85287 USA; 2 Department of Ocean and Mechanical Engineering, Florida Atlantic University, 777 Glades Road, Boca Raton, FL 33431 USA

**Keywords:** Biomedical engineering, Mechanical engineering

## Abstract

Biosample encapsulation is a critical step in a wide range of biomedical and bioengineering applications. Aqueous two-phase system (ATPS) droplets have been recently introduced and showed a great promise to the biological separation and encapsulation due to their excellent biocompatibility. This study shows for the first time the passive generation of salt-based ATPS microdroplets and their biocompatibility test. We used two ATPS including polymer/polymer (polyethylene glycol (PEG)/dextran (DEX)) and polymer/salt (PEG/Magnesium sulfate) for droplet generation in a flow-focusing geometry. Droplet morphologies and monodispersity in both systems are studied. The PEG/salt system showed an excellent capability of uniform droplet formation with a wide range of sizes (20–60 μm) which makes it a suitable candidate for encapsulation of biological samples. Therefore, we examined the potential application of the PEG/salt system for encapsulating human umbilical vein endothelial cells (HUVECs). A cell viability test was conducted on MgSO_4_ solutions at various concentrations and our results showed an adequate cell survival. The findings of this research suggest that the polymer/salt ATPS could be a biocompatible all-aqueous platform for cell encapsulation.

## Introduction

Droplet microfluidics has emerged as a promising technique to generate micrometer-sized emulsions inside microchannels^1–4^. Its immense potential in numerous applications such as chemical reactions^5,6^, material synthesis^7–9^, drug delivery^10–12^ and single-cell encapsulation^13,14^ has been already reported in many publications. Generation of water droplets inside an oil environment named a water-in-oil (W/O) system has been primarily used in droplet microfluidics^4^. However, the biocompatibility issue arising from the toxic nature of some oils could be detrimental to the viability of the biological samples. For example, organic solvents like oil can denature biomolecules such as proteins or enzymes^15^, damage the biological products and formation of tissue, inhibit the cell growth, and accelerate the cell death^16,17^. As a result, an expensive and cumbersome post-processing step is inevitable to remove oil from aqueous droplets containing biological samples (*e.g*. DNA, cells, and proteins) immediately after W/O droplet generation^1,18,19^. To circumvent these challenges in the W/O system, an aqueous two-phase system (ATPS) has been proposed^20^.

ATPS is usually formed by dissolving two incompatible polymers (e.g., polyethylene glycol (PEG)/dextran (DEX)), or a polymer and salt (e.g., PEG/salt) into water. Phase separation occurs above a critical concentration resulting in two aqueous phases each enriched with one of the components^16,20,21^. This system has been widely employed for extraction, separation, purification, and enrichment of biomolecules^16^. ATPS, as an appropriate alternative biocompatible and eco-friendly emulsion to the W/O system, has been also used to produce oil-free and non-toxic aqueous droplets^22^. Despite its early introduction and strong potential, ATPS has gained little attention and lags behind the popularity of the W/O system due to its critical hurdle for droplet generation, which is the difficulty in formation of controlled and stable aqueous droplets in another aqueous phase. This challenge is due mainly to the ultra-low interfacial tension (IFT) of ATPS, which is often less than 10^−1^ mN/m (c.f. 1~40 mN/m in the W/O system), resulting in either a long stream of the dispersed phase throughout the channel without droplet breakup or erratic non-uniform droplets^1,23^. Therefore, applying external forces to actively perturb ATPS is necessary to facilitate the droplet breakup and generation. These methods include mechanical vibration^24^, pulsating pressure^19^, and electrohydrodynamic perturbation^25^. Although active techniques make ATPS droplet generation possible, they are not straightforward and expensive.

On the other hand, passive methods that are much cheaper and simpler than the active methods have been recently reported for ATPS droplet generation^22,23^. The passive ATPS droplets have been primarily generated in a PEG/DEX system^22^ which suffers from the low throughput^22^ or oil involvement in the droplet generation process^23^. Another possibility of ATPS droplet generation is to use a PEG/salt system. The PEG/salt system has been found to possess excellent features such as low cost, short separation time, easier manipulation and disposability^26–28^. More importantly, the PEG/salt system has a relatively higher range of IFT (0.1–1 mN/m) resulting in a faster growth rate of interfacial instability and jet breakup through the Rayleigh-Plateau (R-P) instability when compared with the PEG/DEX system^20^. Due to the high IFT, passive ATPS droplet generation in the PEG/salt system could be possible without applying external perturbations. The high IFT also makes droplet breakup occurred closer to the junction without a long stable jet. Despite the significance and advantages of the PEG/salt system, it received less attention in ATPS droplet generation.

At present, there is no literature regarding the passive salt-based ATPS droplet generation and the biocompatibility analysis of this system. Here, we report passively-generated ATPS droplets in the PEG/salt system. Magnesium sulfate was chosen because it possesses a relatively higher value of interfacial tension at a low concentration when compared with the most available salts. Droplet generation, size, and uniformity in both systems of PEG/salt and PEG/DEX were observed, compared, and analyzed. We found that droplets in the PEG/salt system have a significant better uniformity and a wider range of size controllability when compared with those in the PEG/DEX system. Finally, we tested the biocompatibility of salt-based ATPS droplets for biomedical applications through human umbilical vein endothelial cells (HUVECs) encapsulation. HUVECs that we used are GFP-HUVECs [Green Fluorescent Protein]. GFP molecules fluoresce green color that greatly helps the user study the dynamic changes of cellular processes of living cells in the droplets^29^. Fluorescence from HUVECs also enables us to track the cells conveniently via optical microscopy. Finally, the average size of HUVECs is 14–15 µm which is an ideal size to test the capability of encapsulation by droplets. The viability of the cells in salt media at different concentrations was tested and found that 52% of the cells survived for 2 hours at a 15% salt concentration, showing a good cell viability.

## Experimental Section

### Experimental setup

**Figure 1:**
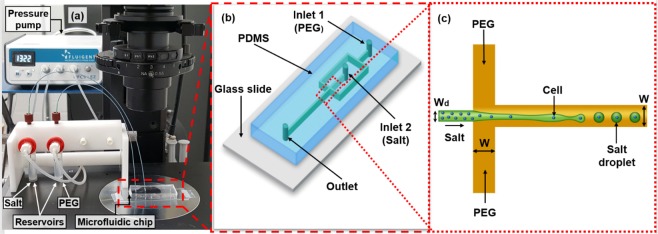
(**a**) Experimental setup including an inverted microscope, a high precision pressure pump, and a microfluidic device. (**b**) Schematic diagram of a microfluidic chip with two inlets and one outlet. (**c**) Schematic diagram of the flow-focusing geometry. Droplets are generated close to the junction and cells are encapsulated inside droplets.

Figure 1 shows the experimental setup for ATPS droplet generation including a microfluidic chip and a high-precision microfluidic pressure control system (MFCS-EZ, Fluigent, Inc., USA). The microfluidic chip consists of a flow-focusing geometry with a height of 50 µm, a dispersed channel width (*W*_*D*_) of 50 µm, and a continuous and main channel width (*W*) of 100 µm. A precise pressure control system includes a pressure pump and two reservoirs for dispersed (salt or DEX) and continuous (PEG) solutions. ATPS droplet generation was observed and recorded using an inverted microscope (IX73, Olympus Corp., Japan) with a 10× objective lens and a high-speed camera (Fastec IL5S, Fastec Imaging Corp., USA). The camera operated at 500 fps with an exposure time of 1.0 ms to capture a series of images at high-speed. ImageJ software was employed for quantification of droplet shapes, droplet sizes, and number of droplets.

### Microfluidic chip

The microfluidic device was fabricated in polydimethylsiloxane (PDMS) using soft lithography and photolithography techniques. Computer-aided design (CAD) software (AutoCAD 2016, Autodesk, Inc., USA) was used to draw channel geometries. The photomask was created by printing the CAD design onto a transparency sheet (25,400 dpi, CAD/ART Services Inc., USA). Then, a layer of KMPR 1025 photoresist (MicroChem, USA) was spin-coated on a 4 inch silicon wafer (UniversityWafer, Inc., USA). After UV exposure and chemical development, the channel pattern was formed. A standard ratio mixture of 10:1 PDMS elastomer base to the crosslinking agent (Sylgard 184, Dow Corning, USA) was poured onto the silicon wafer and baked in the oven for 1 h before removal of the PDMS layer. Inlet and outlet holes were made by punching through the PDMS with a 1.0 mm diameter biopsy punch (Integra Miltex, Inc., Germany). Lastly, oxygen-plasma treatment (Harrick Plasma, USA) was applied for PDMS/glass slide (25 × 75 × 1.0 mm, Fisher Scientific, USA) bonding.

### Chemicals and solution preparation

**Figure 2:**
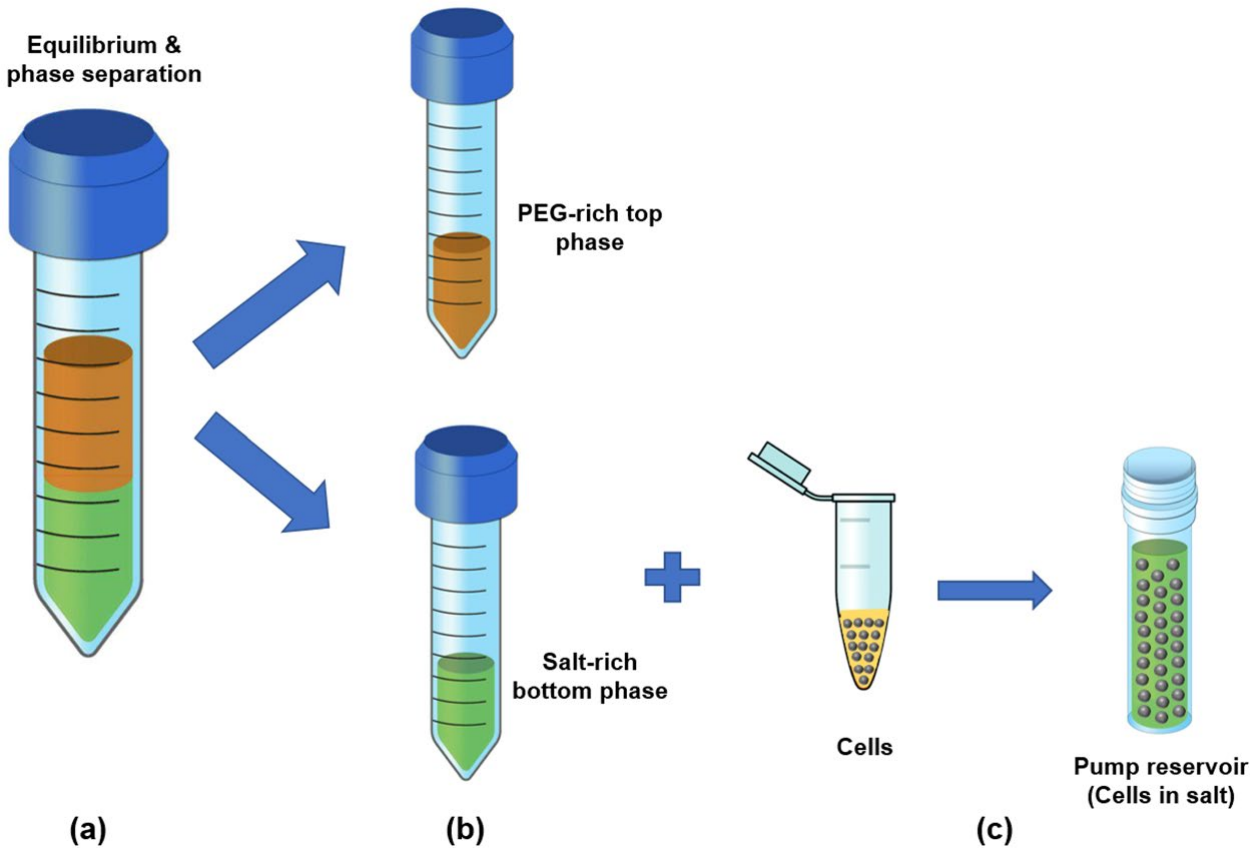
Preparation of ATPS systems for cell encapsulation. (**a**) Mixing of two solutions and phase separation after reaching equilibrium. (**b**) Separation of two phases. (**c**) Addition of cell suspension into the salt medium inside the pump reservoir.

We prepared two types of ATPS including PEG/salt and PEG/DEX systems by dissolving Magnesium sulfate (MgSO_4_) (Sigma-Aldrich, USA), PEG (Sigma-Aldrich, USA) and DEX (Alfa Aesar, USA) separately into deionized water: 7.65% (w/w) Magnesium sulfate, 15.10% (w/w) PEG for the PEG/salt system^30^ and 7.723% (w/w) DEX, 4.827% (w/w) PEG for the PEG/DEX system^21^. Solutions in both systems were vigorously mixed in beakers using magnetic stirrer (Isotemp stirring hotplate, Fisher Scientific) and left for 24 hours (PEG/DEX system) and 8 hours (PEG/salt system) inside two 50 mL conical centrifuge tubes (Corning falcon centrifuge tubes, Fisher Scientific). After reaching equilibrium, phase separation occurs such that the upper is equilibrated with a PEG-rich phase and the lowers are equilibrated with DEX-rich and salt-rich phases (two phases became clear and transparent, and the interface was well-defined). As it is shown in Fig. 2, all equilibrated phases were partitioned by a syringe and transferred to the separate 50 mL conical centrifuge tubes. The IFT of the PEG/DEX system is *σ* = 0.103 mN/m^21^ while it is *σ* = 0.308 mN/m for the PEG/salt system^30^.

### Cell viability

We used 3-[4, 5-dimethylthiazol-2-yl]-2, 5 diphenyltetrazolium bromide (MTT) assay to study the impact of salt at different concentrations on cellular toxicity. HUVECs were grown in a T-75 cell-culture flask with endothelial cell basal medium-2 (EBM-2, Lonza) supplemented with 1% penicillin-streptomycin-glutamine (PSG), 10% fetal bovine serum (FBS), hydrocortisone, EGF, and ascorbic acid (Human epithelial cell medium kit, EGM-2, Clonetics). After obtaining adequate confluence, the cells were detached using Trypsin-EDTA (0.25%) and then were counted using hemocytometer. 5 × 10^3^ cells (400 μl of cell suspension) were cultured on the 24-well plates for 48 hours. Following, the culture medium was replaced with 400 μl of the fresh culture medium containing MgSO_4_ solutions with final nine different concentrations (2.5, 5, 7.5, 10, 12.5, 15, 20, 25, and 30% w/v). After 2, 4, and 6 hours of incubation, we removed the medium from each well and washed them with PBS twice. Then, 40 μl of MTT solution (5 mg/ml) with 360 μl of fresh culture medium was added and the plates were incubated at 37 °C with 5% CO_2_ for 4 hours. After 4 hours, the medium was removed carefully and 400 μl of Dimethylsulfoxide (DMSO) was added to each well in order to solubilize intracellular formosan. The absorbance of collected formosan was recorded at 570 nm with a spectrophotometer (SpectraMax 190 microplate reader). The measurements were carried out in triplicate (n = 3). At the same time, live/dead staining (Live/Dead Kit, Thermo Fisher Scientific) and imaging were carried out on the all nine treated (with 4 hours of incubation time) and control wells using fluorescence microscopy (Nikon Eclipse TE2000-S). For this membrane integrity assay, we added two different fluorescent dyes: Calcein, AM for live cells and ethidium homodimer-1 for dead cells which stain living cells green and dead cells red, respectively. ImageJ was also used for merging the images.

### Statistical analysis

We used one-way ANOVA with Tukey’s post hoc test to compare our different groups. We had three samples in each group and data are shown as a mean ± standard error (SE). The values of p < 0.05 are considered statistically significant.

## Results and Discussion

### ATPS droplet generation in PEG/Salt and PEG/DEX systems

Jetting is the most favorable flow regime to produce droplets with the highest throughput and a wide range of droplet sizes^31^. In this flow regime, the interfacial tension (IFT) between the continuous and dispersed phases plays an important role in determining droplet breakup and generation characteristics. The IFT force attempts to minimize the surface area of the dispersed phase while inducing the Rayleigh–Plateau (R–P) instability. The R-P instability creates breakup of the dispersed jet into multiple droplets at its tip^32^. The high IFT (1~40 mN/m) in the W/O system make droplet formation facile and straightforward, but it is much challenging in APTS due to the intrinsic ultra-low value of IFT (10^-1^ to 10^-4^ mN/m). When the IFT force is very small compared to the effect of shear and inertial forces, it is expected to have a slow growth rate (*ω*) of the instability along the dispersed jet, an increase in droplet breakup length and break time, and a decrease in generation frequency^20^. This behavior can be explained by Eq. 1 that indicates the relationship between the R–P instability growth rate and perturbation wavenumber (*k*), interfacial tension (*σ*), viscosity (*μ*), size of the jet (*r*_0_), and channel height (*h*). In this equation, the lower IFT (*σ*) makes the growth rate (*ω*) slower: in the literature, the slower growth rate makes droplet formation difficult^23,33^. Although a passive method using a pipette tip could generate ATPS droplets in PEG/DEX by decreasing the inlet flow rate significantly^22^, this method is not practical since the input conditions continuously change during droplet formation.

1 $$\begin{array}{rcl}\omega  & = & \frac{1}{8}\frac{\sigma }{{\mu }_{PEG}h}\frac{F(x,\lambda )({k}^{2}-{k}^{4})}{{x}^{9}(1-{\lambda }^{-1})-{x}^{5}}\\ F(x,\lambda ) & = & {x}^{4}(4-{\lambda }^{-1}+4\,\mathrm{ln}\,x)+{x}^{6}(\,-\,8+4{\lambda }^{-1})+{x}^{8}(4-3{\lambda }^{-1}-(4-4{\lambda }^{-1})\mathrm{ln}\,x)\\ \lambda  & = & \frac{{\mu }_{DEX}}{{\mu }_{PEG}},\,x=\frac{{r}_{0}}{(h/2)}\end{array}$$

To enable ATPS droplet generation, two options are possibly considered: one is to facilitate the jet breakup via external means and another is to increase IFT values. A few active methods have been introduced to ease the droplet breakup in the PEG/DEX system, but they are expensive and suffer from a slow droplet generation. As a second option, here we use a PEG/salt system possessing higher IFT than that of the PEG/DEX system. We used a precise pressure pump to inject solutions at low pressures/flow rates to offset the effect of low IFT of ATPS system. In this case, the resultant IFT force could be comparable to the shear force^34^. It should be noted that the IFT of PEG/salt system is (0.1–1 mN/m) when compared to (1~40 mN/m) of the W/O system, which is still a low value.

**Figure 3:**
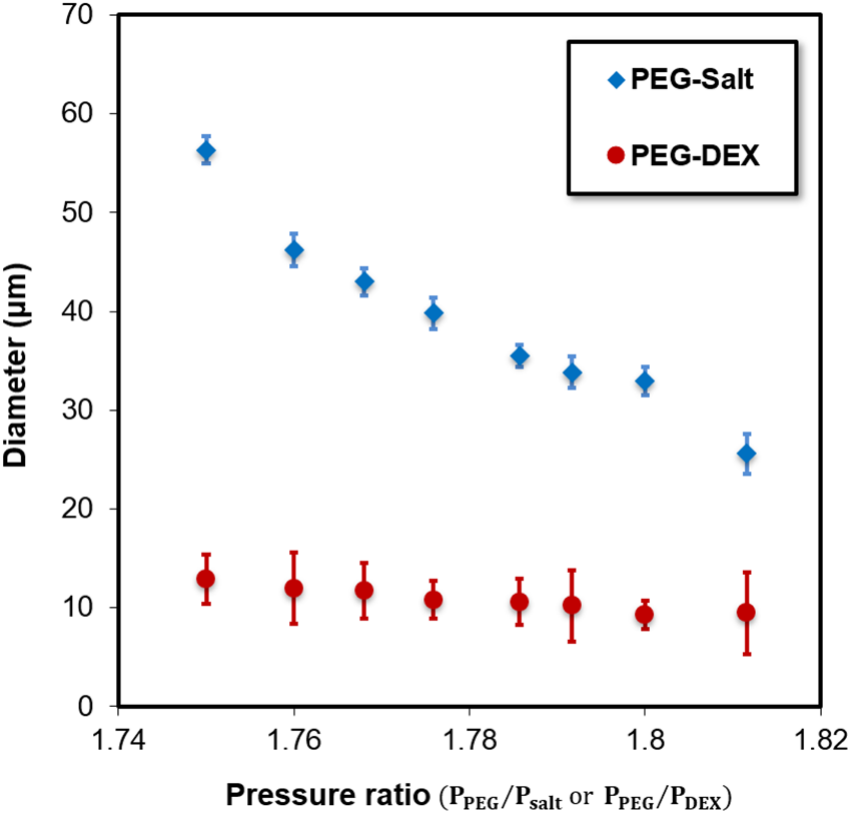
Variations of droplet diameter versus the pressure ratio of the continuous phase (PEG) over dispersed phase (salt and DEX).

For the comparison purpose, we generated ATPS droplets in two systems: PEG/DEX and PEG/salt. To form droplets under the jetting flow regime in both systems, we introduced PEG at 7–20 kPa and salt/DEX at 5–10 kPa. Figure 3 shows variations in a droplet size versus pressure ratio of the continuous phase (PEG) over the dispersed phase (salt/DEX) in both systems. As seen in the figure, the droplet size decreases as the pressure ratio increases in both systems although it is not significant in the PEG/DEX system. This decrease is due to either the injection of PEG at higher pressure or DEX/salt at a lower pressure leading to the smaller droplets. In another word, the inertial and viscous forces of the continuous phase increase with an increase in pressure ratio making a longer and narrower jet and smaller droplets. The size decrease in the PEG/salt system is more pronounced, suggesting that we have a wider range of size selectivity under the given pressure ratio than the PEG/DEX system. The main reason for this difference is the low value of interfacial tension in the PEG/DEX system making unstable tiny droplets. The range of droplet sizes in the PEG/salt system is 25~60 μm while it is 9.2~12.9 μm in the PEG/DEX system. We conjecture that this behavior is attributed to the higher IFT that facilitates stable droplet generation. The error bars represent the standard deviation (SD) and *SD*_*avg*_ = 1.4 µm for the PEG/salt system and *SD*_*avg*_ = 2.8 µm for the PEG/DEX system. The smaller *SD*_*avg*_ in the PEG/salt system suggests that formed salt droplets are more uniform and consistent. For verification, we calculated the average coefficient of variation (CV) in droplet size, defined as the ratio of a standard deviation to the mean of the droplet size, and CV_avg_ = 4% in the PEG-Salt system vs. CV_avg_ = 26% in the PEG/DEX system.

**Figure 4:**
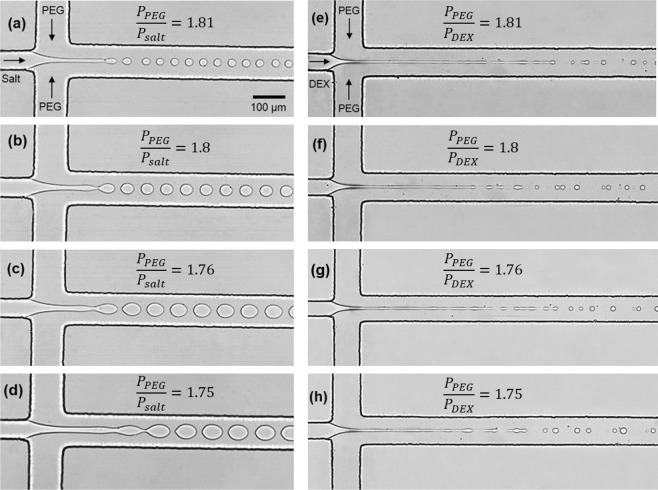
Experimental images of droplet generation in the (**a**–**d**) PEG/salt system; (**e**–**h**) PEG/DEX system. Droplet diameter (*D*) varies with the pressure ratio. (**a**) *D* = 25.5 μm; (**b**) *D* = 32.9 μm; (**c**) *D* = 46.2 μm; (**d**) *D* = 56.3 μm; (**e**) *D* = 9.43 μm; (**f**) *D* = 9.28 μm; (**g**) *D* = 11.97 μm; (**h**) *D* = 12.92 μm.

To characterize droplet generation and size variations in these APTS systems, we performed a high-speed visualization of the sequential droplet breakup process. Figure 4 shows the experimental images of generated droplets in different sizes at different pressures for the PEG/salt (4a-d) and PEG/DEX (4e-h) systems. At given pressure ratios, the breakup length in the PEG/DEX system is longer compared to that in the PEG/salt system due to the lower value of IFT (e.g., 4a vs. 4e). As a result, DEX droplets are generated farther from the junction than salt droplets: 200 μm in 4a and 600 μm in 4e. Although the breakup length in the PEG/DEX system is much longer than in PEG/salt, we found that this length is not correlated with the droplet size.

Figure 4e–h show number of non-uniform tiny satellite droplets as thin DEX jet travels downstream. These tiny droplets are created from the thin thread before the breakup of the main droplets^34^. Under given pressure ratios we tested, the breakup point of the DEX phase was not consistent and occurs at different locations (see Supporting Information Video 1) making inconsistent droplets and thin threads which could have adverse effects on the droplet encapsulation process. It has shown that the existence of small satellite droplets close to the parents droplets in the PEG/DEX system results in poor size distribution of the products and impurities in biochemical tests^35^. This requires using active/passive methods to separate and sort satellite droplets from the main droplets such as using optically induced dielectrophoresis^36^ and double T-junction design^37^ which are expensive and need advanced fabrication processes and equipment. In contrast, no satellite droplets were observed in the PEG/salt system over the pressure ranges tested (see Supporting Information Video 2) and this is clearly seen in Fig. 4a–d, leading to the formation of extremely uniform droplets.

In addition, consistent with Fig. 3, the variations of droplet sizes are more sensitive to the pressure ratio in the PEG/salt system. Figures 4a,e show the smallest droplets at $\frac{{P}_{PEG}}{{P}_{salt/DEX}}=1.81$ while Fig. 4d,h show the largest droplets at $\frac{{P}_{PEG}}{{P}_{salt/DEX}}=1.75$ in both systems: the percent change in a salt droplets size is 60% vs. 28% in DEX droplets. Generation of size-tunable droplets is an important need for many large-scale applications and biological materials of different sizes at a higher manufacturing efficiency while saving significant amounts of chemicals and time^1^. We found that the PEG/salt system conveys high uniform droplets at a wide selectivity under given pressure conditions.

### Cell encapsulation in PEG/Salt ATPS and cell viability

**Figure 5:**
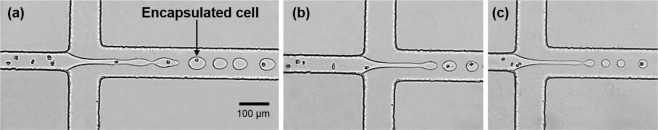
Representative images of cell encapsulation inside salt droplets in different sizes. (**a**) *D* = 42 μm; (**b)** *D* = 34 μm; (**c**) *D* = 25 μm.

The PEG/salt ATPS droplets show excellent uniformity, stability and size controllability. For practical applications of this system, we investigated the capability of passive single-cell encapsulation inside salt droplets as it is the key factor in bio-applications such as single-cell analysis and drug discovery^22^. The HUVECs with a population of 1.5 × 10^6^ cells/mL and an average radius of 14 µm were first centrifuged with a speed of 1000 rpm for 5 min and then were suspended in the salt phase inside the pump reservoir (Fig. 2). Individual cells come to the junction randomly and are encapsulated into the uniform droplets at the end of the jet as a result of passive cell-triggered R-P instability (Fig. 5 and see Supporting Information Video 3). The resulting cell-containing droplets become larger than empty droplets because the breakup location occurs closer to the junction at the same pressure ratio^38^. Figure 5 shows representative images of HUVECs encapsulation in different droplet sizes. Cells were successfully encapsulated and travelled downstream while they stayed inside the salt droplets. We observed that some droplets were empty without cells because the cells come to the junction inconsistently during the droplet formation. It is well known that uniformity in cell encapsulation inside micro-droplets is one of the most challenging issues. Generally, single-cell encapsulation in each microdroplet occurs randomly, resulting in a large number of empty droplets^39^. However, by properly adjusting the inlet flow rates of continuous and dispersed phases and the cell suspension concentration, it could be possible to control the rate of cell encapsulation. In our study, the average rate of encapsulation is about 20–30%.

**Figure 6:**
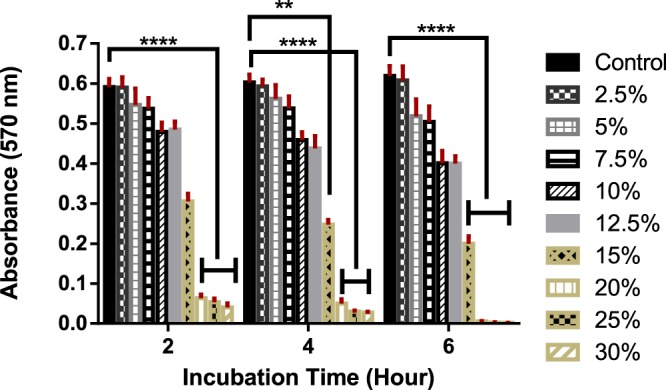
Results of cell viability tests after adding salt solutions at concentrations from 2.5–30% to the cell suspension. MTT assay shows the intensity of absorbance with cell treated time for nine different groups (n = 3).

Finally, we performed the cell viability test off-chip to study the biocompatibility of the MgSO_4_ solution by using cell suspensions in the well-plates and treating the cells at different salt concentrations. Due to the ultralow interfacial tension of ATPS, the generated droplets have weak interfaces that can be torn and broken when they experience a high amount of shear force. This can happen when droplets are drawn by pipette tips to be transferred into the cell viability test environment. As a result, we performed the cell viability test off-chip. Cell proliferation on both the treated and control wells was measured by MTT assay. The MTT measurements are based on the tetrazolium salt reduction to the purple formazan crystals. Figure 6 shows the viability graph of the treated cells and controls. The highest viability is related to control (Tissue Culture Plate) and all the other data are compared with the control data. Therefore, it should be noted that the control group shows 100% viability. In Fig. 6, control means that we cultured the cells on the surface of well-plate and treated them with a culture medium without adding any salts.

**Figure 7:**
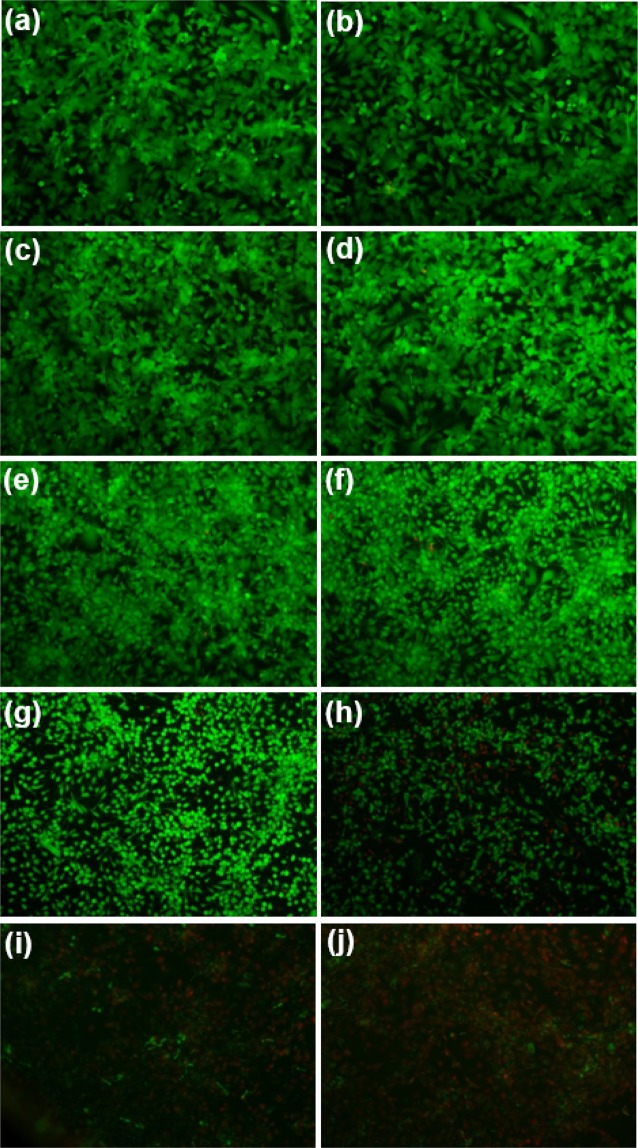
Fluorescence images for morphologies of GFP-tagged Human Umbilical Vein Endothelial cells (HUVECs) on TCP and treated cells with different salt solutions at 10× magnification under fluorescence microscopy. HUVECs have an elongated morphology on the surface of TCP: (**a**) Control; (**b**) 2.5%; (**c**) 5%; (**d**) 7.5%; (**e**)10%; (**f**) 12.5%; (**g**) 15%; (**h**) 20%; (**i**) 25%; (**j**) 30%.

The cells show approximately 99, 92, 90, 81, 81, and 52%, viability after 2 hours of treatment with 2.5, 5, 7.5, 10, 12.5, and 15% w/v of MgSO_4_ solutions, respectively. These results are also around 98, 83, 85, 65, 64 and 32% at the 6 hours mark in MgSO_4_ solutions with a concentration of 2.5, 5, 7.5, 10, 12.5, and 15% w/v, respectively. These data confirm that the cell viability in low concentrations of MgSO_4_ solutions is relatively high while high levels of salt (>15%) enhance the cells killing rate. The salt concentration (7.65% w/w) used for salt-based ATPS droplet generation in this study is ~15% (w/v). The results demonstrate no statistically significant reduction in the cell viability after 2, 4, and 6 hours of treatments with 2.5, 5, 7.5, 10, and 12.5% w/v MgSO_4_ solutions in comparison with the control groups. By increasing the salt concentration more than 15% w/v, a difference does exist after 2 hours of treatment. Base on the results, it is obvious that increasing the salt concentration and exposure time cause an increase in cytotoxicity. Also, from images in Fig. 7, increasing the salt concentration has negative effects on the live cell number, growth rate, and morphology. In addition to the cell viability data, we did not detect any significant cell death in the treated wells after 4 hours under fluoroscopy (Fig. 6). Many types of cell research require the cytotoxicity test over longer periods of time, but the droplets generated in this research are designed to a widespread use in short-term experiments such as genome-sequencing, bioassay, cell assembly, etc.

## Conclusion

Droplet microfluidics offers a wide range of biomedical and bioengineering applications such as high-throughput single-cell assays, DNA sequencing, and protein analysis. In all these applications, successful and efficient encapsulation into droplet is the main step of bioassays. The possibility of passive generation of salt based ATPS microdroplets inside a microfluidic chip with a flow-focusing geometry, cell encapsulation, and its biocompatibility test are successfully reported for the first time in this research. The results were compared with the PEG/DEX system and it was found that the PEG/salt system could result in droplet generation with better properties such as size uniformity, stability and size controllability. The range of droplet sizes in the PEG/salt system is 25~60 μm which is wider compared to 9.2~12.9 μm in the PEG/DEX system. The generate salt droplets are highly monodisperse with CV_avg_ = 4% while it is 26% in the PEG/DEX system. This excellent feature is due mainly to the higher interfacial tension of the PEG/salt system as compared to that in the PEG/DEX system. The PEG/salt system allows the efficient encapsulation of biomaterials in a biofriendly oil-free environment. We tested this feature by encapsulating HUVECs inside the salt droplets. We observed that droplets successfully were transported while they contained cells. Finally, we conducted the cell viability test on a MgSO_4_ solution with different concentrations to examine the biocompatibility of the salt. The test showed a cell viability of more than 50% at salt concentrations of less than 15% w/v.

## Supplementary information

Video1

Video2

Video3
